# *Leishmania infantum-*specific IFN-γ production in stimulated blood from cats living in areas where canine leishmaniosis is endemic

**DOI:** 10.1186/s13071-019-3386-y

**Published:** 2019-03-26

**Authors:** Vito Priolo, Pamela Martínez-Orellana, Maria Grazia Pennisi, Marisa Masucci, David Prandi, Dorotea Ippolito, Federica Bruno, Germano Castelli, Laia Solano-Gallego

**Affiliations:** 1 Dipartimento di Scienze Veterinarie, Università di Messina, Messina, Italy; 2 Departament de Medicina i Cirurgia Animals, Facultat de Veterinària, Universitat Autònoma de Barcelona, Bellaterra, Barcelona, Spain; 3 Centro di Referenza Nazionale per la Leishmaniosi (CReNaL), Istituto Zooprofilattico Sperimentale della Sicilia “A. Mirri”, Palermo, Italy

**Keywords:** Adaptive immunity, Feline, Humoral immunity, IFN-γ release whole blood assay, *Leishmania infantum*, PCR, Retroviral infections

## Abstract

**Background:**

Feline leishmaniosis caused by *Leishmania infantum* is considered a rare disease in endemic areas, whereas subclinical infections are common. Immune response plays a key role in driving the course of *L. infantum* infection in other host species; however, the feline cell-mediated immune response to *L. infantum* infection has not yet been investigated. The aim of this study was to determine the cell-mediated immune response specific to *L. infantum* by means of interferon (IFN)-γ release in whole blood assay from cats living in endemic areas (66 in Sicily and 113 in Catalonia) and to compare with antibody levels to *L. infantum* [enzyme-linked immunosorbent assay (ELISA) and immunofluorescence antibody test (IFAT)], blood parasite load and retroviral infections.

**Results:**

Most cats (*n* = 140) were *L. infantum* antibody negative and only 22% (*n* = 39) were positive. Only 9 and 2% of tested cats had a feline immunodeficency virus (FIV) infection or a feline leukemia virus (FeLV) infection, respectively. Thirty-two cats out of 179 (18%) produced IFN-γ after stimulation with *L. infantum* soluble antigen (LSA) while the majority of cats (93%) produced IFN-γ after stimulation with concanavalin A (ConA). Six LSA-IFN-γ-producer cats were seropositive (three to ELISA and five to IFAT) but they were polymerase chain reaction (PCR) negative, while only one cat was antibody- and PCR-positive. Significant positive correlations were found between IFN-γ concentrations after stimulation with LSA and ConA, and between serology and PCR testing. No association was found between FIV status and LSA or ConA-IFN-γ production. Combining PCR, serology and specific IFN-γ concentration results, we found that 36% of cats studied were exposed to *L. infantum.*

**Conclusions:**

As expected, cats from endemic areas produce IFN-γ after *ex vivo* blood stimulation with LSA and therefore are able to activate a cell-mediated adaptive immune response against the parasite that is variably associated with antibody or blood PCR positivity. The association of this assay to serological and molecular tests provides a better estimate of cat exposure to *L. infantum.*

## Background

Leishmaniosis is a vector-borne disease of humans and animals caused in Europe by *Leishmania infantum* and transmitted by female sand flies of the genus *Phlebotomus* [1–3]. Dogs are considered the main reservoir of *L. infantum* but there is clear evidence that some wild and synanthropic mammals and domestic cats are able to infect sand flies and they play a variable role in a reservoir system according to local and ecological peculiarities [3–5].

Feline leishmaniosis (FeL) due to *L. infantum* infection was described, for the first time, in 1912 by Sergent et al. [6] and since then it has been globally reported in endemic areas [5, 7]. While subclinical feline infections are common in areas where canine leishmaniosis (CanL) is endemic, clinical illness due to FeL is rare [7, 8]. Clinical disease is frequently associated with possible impaired immunocompetence, as in case of retroviral co-infections, immunosuppressive therapy, or malignant neoplasia [7, 8]. The immune response plays a crucial role in the control of *Leishmania* infection. Albeit some differences according to the host species, T cells modulate and orient, through cytokine production, macrophage reaction to the parasite [9–11]. Antigen processing cells (dendritic cells and macrophages) present *L. infantum* antigens to CD4^+^ T cells that modulate the type of immune response [12]. A T helper 1 (Th1) oriented immune response, associated with production of interferon-gamma (IFN-γ), interleukin (IL)-2 and tumor necrosis factor alpha (TNF-α), stimulates phagocytosis by macrophages, their production of nitric oxide and reactive oxygen intermediate and consequent phagocyte-based parasite intracellular elimination [11, 13]. Conversely, in dogs susceptibility to infection and disease progression is mediated predominantly by a non-protective T helper 2 (Th2) immune response and the production of cytokines such as IL-4, IL-10, IL-13 and transforming growth factor beta (TGF-β) which are associated with downregulation of the cellular immune response, a high level of antibodies, and *L. infantum* dissemination [9–11].

Different feline innate and adaptive immune responses might account for the observed lower prevalence of *L. infantum* infection as well as clinical leishmaniosis in cats as compared to dogs [7, 14]. It is well recognized that cats appear to be less frequently affected by arthropod-borne diseases when compared to dogs, although no important differences are known between the canine and feline immune systems [15]. Interestingly, to the best of our knowledge, no studies have so far evaluated *L. infantum*-specific cell-mediated immunity in cats. However, detection of IFN-γ in antigen stimulated whole blood or peripheral blood mononuclear cells (PBMCs) was used in cats to evaluate cellular immune response mechanisms to other pathogens (e.g. feline coronavirus or *Toxoplasma gondii*) [16–18].

The main aim of this study was to determine the *L. infantum*-specific cellular immune response in cats by means of the evaluation of IFN-γ production in stimulated blood from cats living in endemic areas of CanL (Catalonia and Sicily) and correlate it with *L. infantum* antibody levels, blood parasitemia, and retroviral status.

## Methods

### Study areas, cats and sampling

Cats were sampled from March 2016 to April 2017 in two Mediterranean endemic areas of CanL: Sicily (Italy) and Catalonia (Spain). Catalan samples were collected at the Fundació Hospital Clínic Veterinari (Bellaterra, Barcelona), Hospital Clinic Xinesca (Vilassar de Mar, Barcelona) and Vetamic Hospital Veterinari Cambrils (Cambrils, Tarragona). Sicilian samples were collected at Ospedale Veterinario Didattico (Università degli Studi di Messina, Dipartimento di Scienze Veterinarie, Messina) and at Ambulatorio Veterinario Santa Lucia (Lipari, Messina). Inclusion criteria for enrollment included the exposure to at least one sand-fly season and no treatment with repellent ectoparasiticides (i.e. pyrethroid products). Sex, age class, breed, clinical status, lifestyle of pet cats (indoors, outdoors), and number of stray cats at each sampling site are summarized in Table 1. Age was classified as follows: young (6–18 months); adult (between 19 and 96 months); and old (> 96 months). The clinical status of cats was defined as “sick” or “apparently healthy” based on data available from history and physical examination. Sick cats were considered when clinical signs compatible with FeL (i.e. lymph node enlargement, skin, mucosal or eye lesions, stomatitis, weight loss, chronic kidney disease, anaemia) were found as previously described [7]. Apparently healthy cats were considered when no clinical signs were present with the exception of four cats admitted because of trauma that were also included in this group.

**Table 1:** Age class, sex, breed, lifestyle and clinical status of cats studied according to their geographical distribution

Provenience (*N*; %)	Age class			Sex		Breed		Category^a^			Clinical status	
	Young *n* (%)	Adult *n* (%)	Old *n* (%)	Male *n* (%)	Female *n* (%)	DSH *n* (%)	Pure breed *n* (%)	Indoor *n* (%)	Outdoor *n* (%)	Stray *n* (%)	Apparently healthy *n* (%)	Sick *n* (%)
Sicily												
Messina City (*N* = 24; 36)	6 (25)	18 (75)	0 (0)	9 (38)	15 (62)	24 (100)	3 (12)	3 (12)	5 (21)	16 (67)	11 (46)	13 (54)
Aeolian Islands (*N* = 42; 64)	9 (21)	26 (62)	7 (17)	19 (45)	23 (55)	41 (98)	1 (2)	1 (2)	40 (96)	1 (2)	19 (45)	23 (55)
Total (*N* = 66; 37)	15 (23)	44 (67)	7 (10)	28 (42)	38 (58)	65 (99)	1 (1)	4 (6)	45 (68)	17 (26)	30 (46)	36 (54)
Catalonia												
Vilassar de Mar (*N* = 22;19)	11 (50)	7 (32)	4 (18)	13 (59)	9 (41)	17 (77)	5 (23)	7 (32)	11 (50)	4 (18)	17 (77)	5 (23)
Cambrils (*N* = 45; 40)	3 (7)	40 (89)	2 (4)	18 (40)	27 (60)	42 (93)	3 (7)	0 (0)	0 (0)	45 (100)	40 (89)	5 (11)
Bellaterra (*N* = 46; 41)	6 (13)	39 (85)	1 (2)	25 (54)	21 (46)	44 (96)	2 (4)	0 (0)	15 (33)	31 (67)	32 (70)	14 (30)
Total (*N* = 113; 63)	20 (18)	86 (76)	7 (6)	56 (49)	57 (51)	103 (91)	10 (9)	7 (6)	26 (23)	80 (71)	89 (79)	24 (21)
All cats (*N* = 179; 100)	35 (19)	130 (73)	14 (8)	84 (47)	95 (53)	168 (94)	11 (6)	11 (6)	71 (40)	97 (54)	119 (66)	60 (34)

^a^Indoor and outdoor columns concern the lifestyle of pet cats

*Abbreviation*: DSH, domestic short-hair

One milliliter of blood, aseptically put into a heparin tube, was used for whole blood assay within 24 h after blood sampling. EDTA blood and blood serum were also obtained and immediately aliquoted and stored at -20 °C until processed for DNA extraction and serological investigations, respectively.

### Feline IFN-γ release whole blood assay

Whole blood assays were performed as previously described in dogs [10] with some minor modifications to adapt the technique to the smaller volume of blood available. Heparinized blood was diluted at a ratio of 1:10 with medium and after five days the supernatant was collected from unstimulated cultured cells. Two aliquots of diluted blood were respectively cultured with Concanavalin-A (ConA) or *L. infantum* soluble antigen (LSA) to subsequently recover supernatants from stimulated blood cells.

Feline IFN-γ concentrations were determined using a specific DuoSet® ELISA (Development Sistems R&D™, Abingdon, UK) according to the manufacturer’s instructions with some modifications. The standard curve for IFN-γ started with 4000 pg/ml and serial 2-fold dilutions were made until a concentration of 31.25 pg/ml was reached. Duplicates of supernatants obtained from whole blood culture were tested in the ELISA plates. The optical density was measured with an ELISA reader (Anthos 2020, Cambridge, UK) at wavelength of 450 nm. The standard curve was calculated using a computer generating four parameters logistic curve-fit with the program MyAssays (http://www.myassays.com/). Cats were classified as IFN-γ producers (IFNγ-p) when the *L. infantum*-specific IFN-γ concentration, after subtracting the value obtained with supernatant of unstimulated cell cultures, was higher than the last detectable dilution of the standard curve (31.25 pg/ml). Similarly, cats were classified as IFN-γ non-producers (IFNγ-np) when the *L. infantum*-specific IFN-γ concentration, after subtracting the value obtained with supernatant of unstimulated cell cultures, was less than 31.25 pg/ml or at undetectable levels. The same cat classification was made for IFN-γ production obtained by ConA stimulation of whole blood cultures.

### *Leishmania infantum* antibody detection

#### IFAT

Anti-*L. infantum* IgG antibodies were detected using the *L. infantum* (strain MHOM/IT/80/IPT1) antigen produced by C.Re.Na.L. (Centro di Referenza Nazionale per la Leishmaniosi, Palermo, Italy). Fluoresceinated anti-cat immunoglobulin G (IgG) antibody (working anti-feline IgG (H+L)-FITC, Fuller Laboratories, Fullertone, CA, USA) was used. The manufacturer’s protocol was followed, and the end point titer of positive samples was determined preparing serial 2-fold dilutions of serum starting from 1:20. The cut-off value for positivity was set at 1:80 [19, 20]. Fluorescence microscope readings were made by a unique operator (MM).

#### In-house ELISA

ELISA was performed as previously described [20, 21]. All plates included the serum from a sick cat from Cyprus with a confirmed infection with *L. infantum* as positive control, and the serum of a cat from an area where leishmaniosis was not endemic as a negative control. All samples were run in duplicate. The cut-off was established at 12.3 ELISA units (EU) (mean ± 3 standard deviations of sera from 81 cats from the UK, a non-endemic area).

### Detection of anti-FIV antibodies and FeLV p27 antigen

Due to insufficient amounts of serum, only 149 cats out of 179 were tested for feline immunodeficiency virus (FIV) and 171 for feline leukemia virus (FeLV) infections. A total of 133 feline sera were tested for the detection of FeLV p27 antigen and anti-FIV antibodies by a rapid ELISA (SNAP Combo Plus FeLV antigen and FIV antibody test, Idexx Laboratories, Westbrook, ME, USA), according to the manufacturer’s protocol. Because of serum paucity, other cats were tested by a commercial ELISA: 38 serum samples were tested for FeLV p27 antigen by INgezim-FeLV DAS (Ingenasa, Madrid, Spain) and only 16 feline sera for anti-FIV antibodies by INgezim-FIV (Ingenasa).

### Blood DNA extraction and *Leishmania* real-time PCR

Total DNA was extracted from EDTA blood using the DNA Gene extraction kit (Sigma Aldrich, Saint Louis, MO, USA) following the manufacturer’s instructions with some modifications. Forty microliters of proteinase K solution were added to all samples. Four hundred microliters of whole blood were used for all the samples. Blood from a clinically healthy non-infected cat was used as a control for DNA contamination in every DNA extraction performed. The real-time polymerase chain reaction (RT-PCR) was carried out in a CFX96 Real-time System (Bio-Rad Laboratories s.r.l., Hercules, CA, USA) using TaqMan Master Mix (Applied Biosystems by ThermoFisher, Waltham, MA, USA) and performed as previously described [22].

### Statistical analysis

A minimum sample size of 173 cats was calculated for cat enrollment, based on the prevalence of feline *L. infantum* infection in areas under study (Catalonia and Sicily) [14, 19, 20, 23], and on the assumptions of 99% confidence level and 5% precision [24].

Fisher’s exact test was used to compare groups defined by categorical variables. Results of tests for *L. infantum* exposure of cats did not pass the D’Agostino-Pearson normality test. Accordingly, the Mann-Whitney U-test was used to compare unmatched continuous data and the Wilcoxon signed-rank test to compare paired continuous variables. Spearman’s correlation coefficient was calculated to evaluate relationships between ELISA, IFAT and PCR results and between levels of IFN-γ, anti-*Leishmania* antibodies, and *L. infantum* DNA in blood of cats studied. *P*-values<0.05 were considered significant. The D’Agostino-Pearson normality test and Fisher’s exact test were performed using Prism 7 for Mac IOS and all other tests were performed using SPSS software v.17.0 for Windows. Finally, Cohen’s kappa coefficient was measured analyzing results of ELISA and IFAT with blood PCR (https://idostatistics.com/cohen-kappa-free-calculator).

## Results

### Cats

Sex, breed, age class, lifestyle, origin (region and municipality) and clinical status of 179 cats included in the study are summarized in Table 1. The median of age of cats was 24 months [25–75 percentile (25–75) = 18–48 months; interquartile range (IQR) = 30 months]. There were no differences between the two regions related to the variables sex, breed, age class and lifestyle of cats. Conversely, cats from Sicily were more frequently found sick compared to Catalan cats (Fisher’s exact test, *P* < 0.0001).

### *Leishmania infantum* serological tests

**Fig. 1:**
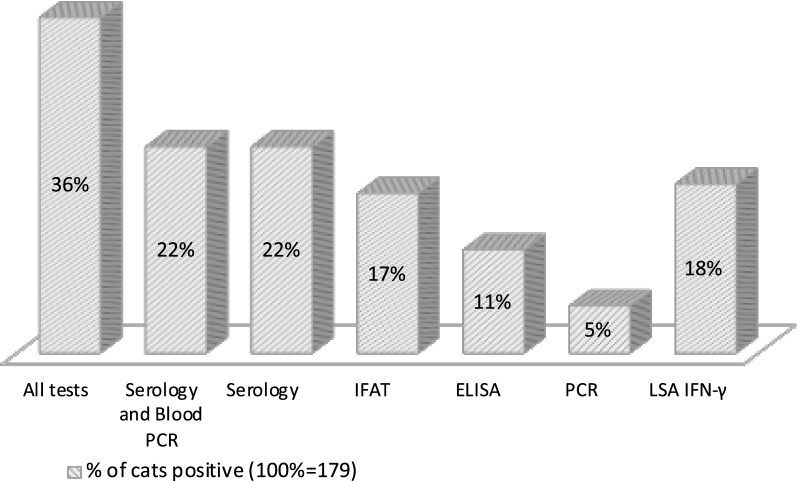
Percentage of cats positive to *L. infantum* according respectively to: all specific tests performed, serology (IFAT and/or ELISA) and blood PCR, serology (IFAT and/or ELISA), IFAT, ELISA, blood PCR, and LSA IFN-γ assay

Results of serological tests are shown in Table 2 and Fig. 1. A slight positive correlation (Spearman’s correlation coefficient, *r*_*s *_= 0.342, *P *= 0.0001) was found between ELISA and IFAT. However, only 11 of the 39 cats positive to IFAT and/or ELISA were positive to both tests (28%) and an agreement of 78.1% was found when these tests were compared (Cohen’s kappa coefficient = 0.18, i.e. slight agreement beyond chance). No differences were found regarding seropositivity and variables studied.

**Table 2:** Number and percentage of cats positive to *L. infantum* and retroviruses according to their geographical distribution and clinical status

Provenience or clinical status (*N*; %)	Serology			Blood PCR	IFN-γ assay		Retroviruses	
	Total	ELISA	IFAT		LSA	ConA	FIV	FeLV
Sicily								
Messina City (*N* = 24; 36)	5 (21)	4 (17)	3 (12)	1 (4)	6 (25)	24 (100)	2 (8)	0 (0)
Aeolian Islands (*N* = 42; 64)	9 (21)	7 (17)	6 (14)	2 (5)	5 (12)	39 (93)	4 (9)	0 (0)
Total (*N* = 66; 37)	14 (21)	11 (17)	9 (14)	3 (4)	11 (17)	63 (95)	6 (9)	0 (0)
Catalonia								
Vilassar de Mar (*N* = 22;19)	1 (4)	0 (0)	1 (4)	1 (4)	2 (9)	22 (100)	1 (5)	0 (0)
Cambrils (*N* = 45; 40)	8 (18)	2 (4)	6 (13)	1 (2)	5 (11)	40 (89)	4 (12)	1 (2)
Bellaterra (*N* = 46; 41)	16 (35)	6 (13)	15 (33)	4 (9)	14 (30)	41 (89)	2 (6)	3 (7)
Total (*N* = 113; 63)	25 (22)	8 (7)	22 (19)	6 (5)	21 (19)	103 (91)	7 (8)	4 (4)
Apparently healthy (*N* = 119; 66)	21 (18)	6 (5)	19 (16)	3 (2)	21 (18)	108 (91)	8 (7)	2 (2)
Sick (*N* = 60, 34)	18 (30)	13 (22)	12 (20)	6 (10)	11 (18)	58 (97)	5 (8)	2 (3)
All cats (*N* = 179; 100)	39 (22)	19 (11)	31 (17)	9 (5)	32 (18)	166 (93)	13 (9)	4 (2)

*Abbreviations*: FIV, feline immunodeficiency virus; FeLV, feline leukemia virus; LSA, *Leishmania* soluble antigen; ConA, concanavalin A

### IFAT

IFAT results based on cat provenience and clinical status are summarized in Table 2. The median IFAT titer was 80 [(25–75) = 80–320]. No differences of IFAT titers were found between Sicilian and Catalonian cats. However, Catalonian cats from Bellaterra were more frequently found positive to IFAT than those from Vilassar de Mar (Fisher’s exact test, *P* = 0.0453).

### ELISA

ELISA results based on cat provenience and clinical status are summarized in Table 2. The median value of positive samples was 19 EU [(25–75) = 13.5–107.3 EU]. Old cats (4/14, 29%) were more frequently positive than young cats (1/35, 3%) (Fisherʼs exact test, *P* = 0.0194). Sicilian cats were statistically more frequently found positive to ELISA than Catalan cats (Fisher’s exact test, *P* = 0.0282) and the ELISA median value for Sicilian cats was significantly higher [6.1 EU; (25–75) = 1.2–9.5 EU] than the result observed in Catalan cats [median: 3.7 EU; (25–75) = 1.9–6.4 EU] (Mann-Whitney U-test, *Z* = -249, *P *< 0.0001).

### *Leishmania* real-time PCR

PCR results based on cat provenience and clinical status are summarized in Table 2. Only 9 cats (5%) were *Leishmania*-PCR positive (Fig. 1) and the median parasite load was 60 *L. infantum* amastigotes/ml [(25–75) = 10–128.5]. *Leishmania*-PCR positive cats were more frequently positive by serology (89% by IFAT and 78% by ELISA) than *Leishmania-*PCR negative cats (13% by IFAT and 7% by ELISA) (Fisher’s exact test, *P*<0.0001). In addition, antibody level measured by IFAT (Mann-Whitney U-test, *Z* = 86.5, *P *< 0.0001) and ELISA (Mann-Whitney-U test, *Z* = 219, *P *< 0.0001) of *Leishmania-*PCR positive cats was significantly higher when compared to *Leishmania-*PCR negative cats. Moreover, a positive correlation was found between PCR and ELISA (Spearman’s correlation coefficient, *r*_*s*_ = 0.272, *P* = 0.0001) with an agreement of 86.1% (Cohen’s kappa coefficient = 0.25, i.e. fair agreement) as well as a positive correlation between PCR and IFAT (Spearman’s correlation coefficient, *r*_*s*_ = 0.347, *P* = 0.0001) with an agreement of 80.1% (Cohen’s kappa coefficient = 0.18, i.e. slight agreement). No other differences were found in positive PCR prevalence according to variables under study.

### Anti-FIV antibodies and FeLV p27 antigen

Thirteen cats (9%) were antibody-positive to FIV and 4 (2%) were antigen-positive to FeLV (Table 2). FIV and FeLV co-infections were never detected. All FeLV-positive cats were from Catalonia while there was no difference in FIV prevalence between cats enrolled in Sicily (9%) and in Catalonia (8%) (Table 2). Moreover, there were no statistical differences in prevalence of anti-*L. infantum* antibodies or *Leishmania* DNA positivity in cats positive to FIV or FeLV compared to the negatives. Similarly, no statistical difference was found for the levels of anti-*L. infantum* antibodies or *L. infantum* parasite load between FIV- or FeLV-positive cats and the negative ones. Interestingly, four FIV- and two FeLV-positive cats were also positive to one or more *L. infantum* diagnostic tests. Only one FeLV- and two FIV-positive cats produced IFN-γ after LSA stimulation. Moreover, two of them were positive to both serological tests and blood PCR. Conversely, 107 cats were *L. infantum* and retrovirus negative and 21 produced IFN-γ after LSA stimulation. There were no statistical differences in IFN-γ productions (after LSA or ConA stimulations) between FIV- or FeLV-positive and negative cats.

### IFN-γ production

The frequency of cats producing IFN-γ after stimulation with LSA or ConA is summarized in Table 2 according to their provenience and clinical status. A higher number of cats produced IFN-γ after stimulation with ConA (166/179, 93%) than with LSA (32/179, 18%) (Fisher’s exact test, *P *< 0.0001) (Table 2). Levels of IFN-γ according to serological and PCR results, and clinical status are summarized in Table 3. The median of the IFN-γ concentration of LSA-IFN-γ producer cats (IFNγ-p) was significantly lower than concentration obtained with ConA stimulation [1115 pg/ml (25–75) = 199.9–2931 pg/ml] (Wilcoxon signed-rank test, *Z* = -11.108, *P* < 0.0001). The unique IFNγ-p cat positive to *Leishmania*-PCR (125 *L. infantum* amastigotes/ml) tested high positive by both IFAT (titer 40960) and ELISA (> 346.3 EU). This cat was adult, FeLV-positive, suffered from squamous cell carcinoma and *Leishmania* amastigotes were detected at cytological evaluation of the neoplastic cutaneous lesion.

**Table 3:** Concentrations of IFN**-**γ and level of positivity to *L. infantum* tests according to results of tests and the clinical status in IFN-γ producer and non-producer cats

*L. infantum* tests or clinical status	No. of cats (%)	LSA IFN-γ (pg/ml)^a^	ConA IFN-γ (pg/ml)^a^	ELISA (EU)^a^	IFAT (titer)^a^	PCR (amastigotes/ml)^a^
IFN-γ producer cats						
Serology- and PCR-negative	25 (14)	174 (64.2; 659.9)	2280 (500.7; 4068)	3.4 (2.1; 6.8)	20 (0; 40)	0 (0; 0)
Serology-positive and PCR-negative	6 (3)	193.9 (103.4; 685.3)	4456 (1756; 5734)	8.9 (1.4; 16.1)	80 (70;140)	0 (0; 0)
Serology- and PCR-positive	1 (1)	276.3	2083.9	> 346.3	40960	125
Apparently healthy	21 (18)	116 (63.7; 238.9)	2097 (205.7; 3976)	3.2 (1.4; 6.1)	20 (0; 40)	0 (0; 0)
Sick	11 (18)	567.1 (276.3; 1747)	3509 (2326; 4967)	6.9 (4.1; 12.7)	20 (0; 80)	0 (0; 0)
Total	32 (18)	203.5 (65.9; 586.7)	2360 (979.1; 4297)	3.7 (2.1; 8.1)	20 (0; 40)	0 (0; 0)
IFN-γ non-producer cats						
Serology- and PCR-negative	115 (64)	0 (0; 0)	646.1 (137.9; 2279)	4.2 (2.4; 6.3)	20 (0; 40)	0 (0; 0)
Serology-positive and PCR-negative	24 (13)	0 (0; 0)	803.2 (176; 2553)	7.65 (4.7; 14.2)	80 (50; 160)	0 (0; 0)
Serology- and PCR-positive	8 (4)	0 (0; 0)	1334 (303.8; 2370)	25.5 (9.7; 263.5)	320 (80; 1120)	60 (7.5; 170)
Apparently healthy	98 (82)	0 (0; 0)	587.5 (127.4; 2449)	4.3 (2.6; 6.7)	40 (0; 40)	0 (0; 0)
Sick	49 (82)	0 (0; 0)	1019 (204.7; 2219)	5.8 (3.0; 8.9)	20 (10; 40)	0 (0; 0)
Total	147 (82)	0 (0; 0)	742.5 (150.7; 2407)	4.5 (2.8; 7.2)	40 (0; 40)	0 (0; 0)

^a^Median values (25th and 75th percentiles)

Sick and apparently healthy cats had the same prevalences of IFNγ-p individuals (Table 2); however, sick IFNγ-p cats had a significantly higher level of LSA IFN-γ (Mann-Whitney U-test, *Z* = 47, *P* = 0.0056) (Table 3). The prevalence of cats producing IFN-γ after stimulation with ConA was above 90% in both groups (Table 2); however, among 64 cats exposed to *L. infantum* (i.e. positive to at least one specific test), sick individuals (*n* = 25) had a significantly higher median concentration [2159 pg/ml; (25–75) = 965.3–4508 pg/ml] than apparently healthy cats (*n* = 39) [1712.5 pg/ml; (25–75) = 87.1–2946 pg/ml] (Mann-Whitney U-test, *Z* = 339, *P* = 0.0117). The concentration of IFN-γ produced after stimulation with ConA was significantly higher in the IFNγ-p group (Mann-Whitney U-test, *Z* = 1473, *P* = 0.0008) (Table 3) and IFN-γ concentrations after ConA or LSA stimulations were positively correlated (Spearman’s correlation coefficient, *r*_*s*_ = 0.264, *P* = 0.0001). Additionally, ELISA antibody levels were positively correlated with IFN-γ concentrations from ConA stimulated cultures (Spearman’s correlation coefficient, *r*_*s*_ = 0.209, *P* = 0.0001).

### Overall *L. infantum* frequency of infection

An overall *L. infantum* prevalence of 36% calculated by PCR, serology and LSA IFN-γ assay was obtained in the population studied (Fig. 1) and no differences were found between Catalonia (35%) and Sicily (36%), the sites of each region studied, or between apparently healthy (33%) and sick cats (42%).

## Discussion

To our knowledge, this study demonstrated for the first time that cats naturally exposed to *L. infantum* infection produced IFN-γ after *ex vivo* whole blood stimulation with *L. infantum* antigens, as occurs in dogs, humans and laboratory animals [10, 25, 26].

Parasite-specific IFN-γ production was found in 18% of enrolled cats and it was associated with antibody production in only seven of the 32 IFNγ-p cats of this study. Both types of adaptive immune responses were therefore variably combined in single cats and a wide immunological spectrum may exist also in cats as already reported in dogs and humans [10, 27].

The present study did not aim to evaluate the parasite T cell mediated immunity of clinical cases of FeL but based on data available from physical examination, the frequency of IFNγ-p individuals was not different in apparently healthy and sick cats. Similarly, there was no difference in the prevalence of sick cats between IFNγ-p and IFNγ-np cats positive to serological or blood PCR tests. However, sick IFNγ-p cats produced a significantly higher level of IFN-γ. Moreover, the unique cat with clinical FeL confirmed by cytology was IFNγ-p and, despite possible immunosuppression due to FeLV infection and to neoplasia, this cat reached an IFN-γ level around the median value. In the present study, retroviral infection *per se* did not affect adaptive immune response of cats to *L. infantum*, but a limitation was the very low number of FIV (*n* = 13) or FeLV (*n* = 4) infected enrolled cats.

Dogs with mild or moderate CanL produce IFN-γ and the lack of production is restricted to severe disease [10, 28]. Prospective clinical studies with cats with confirmed clinical leishmaniosis are needed to further evaluate if IFN-γ is as possible marker for staging the severity of disease and evaluating efficacy of therapy as documented in dogs [10, 28].

IFN-γ production after stimulation with ConA was obtained in almost all cats and it was not associated with any variable, including clinical status, retroviral infections or exposure to *L. infantum*. These findings are similar to data from studies of CanL [10, 28]. As expected, concentrations of ConA-IFN-γ were significantly higher than LSA-IFN-γ and they were positively correlated. Production of ConA-IFN-γ suggests non-specific T cell activation. Surprisingly, when considering cats exposed to *L. infantum* (i.e. positive to at least one the specific tests performed), sick individuals produced a significantly higher level of ConA compared to apparently healthy individuals. This finding is unusual, and it is difficult to interpret due to the limitations of a clinical evaluation only based on physical examination and clinical history. In dogs with CanL, ConA-IFN-γ concentrations do not vary significantly in different LeishVet clinical stages, unless in the severe stage of disease (LeishVet stage IV) when a significantly lower concentration is observed [10].

The prevalence of anti-*L. infantum* antibodies obtained in this study by combination of both IFAT and ELISA results was similar in the two regions with an approximate value of 20%. It is not easy, therefore, to explain the higher frequency and level of positivity of ELISA in Sicilian cats but these cats were more frequently found sick compared to Catalan cats (Table 2). Previous studies conducted in Sicily were based on IFAT and, when the same cut-off was used, anti-*L. infantum* antibody prevalence ranged between 6.6–29% [19, 29–32]. Conversely, only two studies evaluated anti-*L. infantum* antibody prevalence in Catalonia; they both used ELISA and antibody prevalence was 1.7 and 5.3%, respectively [14, 33]. However, as already reported, we found a positive correlation between ELISA and IFAT [21].

The parasite load of cats was measured by blood PCR, thus some positive cats were possibly missed because blood is not the most sensitive tissue for the detection of *L. infantum* DNA in dogs and likewise with cats [7, 34]. However, all PCR-positive cats were also antibody-positive and the level of *L. infantum* DNA in blood was positively correlated with the antibody level of cats. This is not a common finding in cats from *L. infantum* endemic areas but, compared to other studies, we used two serological techniques and therefore the sensitivity of serology was increased [19, 20, 35].

In this study, most IFNγ-p cats were negative to *L. infantum* antibody and DNA detection in blood (25/32, 78%). This finding means that the combination of serological and molecular tests with *L. infantum*-specific IFN-γ evaluation offered a more accurate overall estimation of the exposure to *L. infantum* of cats under study. In fact, the prevalence of *L. infantum* was 5% for blood PCR and 22% for antibody detection but the combination of results obtained by serological and molecular testing did not increase the percentage of positivity because all PCR positive cats were also positive for *L. infantum* antibodies (Fig. 1). However, when also considering positivity for *L. infantum*-specific IFN-γ production, the overall *L. infantum* prevalence raised to 36%. These data confirm that a considerable percentage of the studied cats had contact with *L. infantum* and that detection of cell-mediated immune response by measuring specific IFN-γ production provides a better estimation of exposure of cats to *L. infantum* in endemic areas as seen in dogs with different techniques [34, 36].

## Conclusions

As expected, cats from endemic areas produce IFN-γ after *ex vivo* blood stimulation with LSA and therefore are able to activate a cell-mediated adaptive immune response against the parasite that is variably associated with antibody or blood PCR positivity. The association of this assay to serological and molecular tests provides a better estimate of cat exposure to *L. infantum.*

## Abbreviations

CanL: canine leishmaniosis
ConA: concanavalin A
ConA-IFNγ: IFN-γ produced in whole blood assay after stimulation with ConA
DTH: delayed type hypersensitivity reaction
ELISA: enzyme-linked immunosorbent assay
FeL: feline leishmaniosis
FeLV: feline leukemia virus
FIV: feline immunodeficiency virus
IFAT: indirect fluorescence antibody test
IFN-γ: interferon-γ
IFNγ-np: cats IFN-γ non-producers in whole blood assay after stimulation with LSA
IFNγ-p: cats IFN-γ producers in whole blood assay after stimulation with LSA
IgG: immunoglobulin G
LPA: lymphocyte proliferation assay
LSA: *Leishmania* soluble antigen
PBMCs: peripheral blood mononuclear cells
RT-PCR: real-time polymerase chain reaction
